# Paraneoplastic limbic encephalitis as a cause of new onset of seizures in a patient with non-small cell lung carcinoma: a case report

**DOI:** 10.1186/1752-1947-2-270

**Published:** 2008-08-13

**Authors:** Vasileios Voutsas, Efrosyni Mylonaki, Konstantinos Gymnopoulos, Athanasios Kapetangiorgis, Christos Grigoriadis, Styliani Papaemanuell, Evaggelos Vafiadis, Pandora Christaki

**Affiliations:** 1 2nd Department of Pulmonary Medicine, 'G. Papanikolaou' General Hospital, Exohi Thessalonikis, PC 57010, Greece; 2 Neurology Department, General Clinic 'Ag. Lukas', Panorama Thessalonikis, PC, 55236, Greece; 3 Neurosurgery Clinic, 'G. Papanikolaou' General Hospital, Exohi Thessalonikis, PC, 57010, Greece; 4 Pathology Department, 'G. Papanikolaou' General Hospital, Exohi Thessalonikis, PC, 57010, Greece; 5 Department of Computed Tomography and Ultrasonography, 'G. Papanikolaou' General Hospital, Exohi Thessalonikis, PC, 57010, Greece

## Abstract

**Introduction:**

The etiology of seizure disorders in lung cancer patients is broad and includes some rather rare causes of seizures which can sometimes be overlooked by physicians. Paraneoplastic limbic encephalitis is a rather rare cause of seizures in lung cancer patients and should be considered in the differential diagnosis of seizure disorders in this population.

**Case presentation:**

This case report describes the new onset of seizures in a 64-year-old male patient receiving chemotherapy for a diagnosed stage IV non-small cell lung carcinoma. After three cycles of therapy, he was re-evaluated with a chest computed tomography which showed a 50% reduction in the tumor mass and in the size of the hilar and mediastinal lymphadenopathy. Twenty days after the fourth cycle of chemotherapy, the patient was admitted to a neurological clinic because of the onset of self-limiting complex partial seizures, with motionless stare and facial twitching, but with no signs of secondary generalization. The patient had also recently developed neurological symptoms of short-term memory loss and temporary confusion, and behavioral changes. Laboratory evaluation included brain magnetic resonance imaging, magnetic resonance spectroscopy of the brain, serum examination for 'anti-Hu' antibodies and stereotactic brain biopsy. Based on the clinical picture, the patient's history of lung cancer, the brain magnetic resonance imaging findings and the results of the brain biopsy, we concluded that our patient had a 'definite' diagnosis of paraneoplastic limbic encephalitis and he was subsequently treated with a combination of chemotherapy and oral steroids, resulting in stabilization of his neurological status. Despite the neurological stabilization, a chest computed tomography which was performed after the 6th cycle showed relapse of the disease in the chest.

**Conclusion:**

Paraneoplastic limbic encephalitis is a rather rare cause of new onset of seizures in patients with non-small cell lung carcinoma. Incidence, clinical presentation, laboratory evaluation, differential diagnosis, prognosis and treatment of this entity are discussed.

## Introduction

The etiology of seizure disorders in patients with cancer is broad. Intracranial metastasis, adverse drug reactions, drug withdrawal or intoxication, metabolic disturbances and infections are the most common causes, but the differential diagnosis also includes rarer causes which can sometimes be overlooked by physicians treating such patients. We report a case of paraneoplastic limbic encephalitis (PLE) which is a rather rare cause of seizures in patients with non-small cell lung carcinoma.

## Case presentation

Stage IV (T_4_N_2_M_0_) undifferentiated large cell lung carcinoma was diagnosed in a 64-year-old Greek man. He was a smoker with a smoking history of 60 pack-years. Twenty-two years earlier, he had been diagnosed with a seminoma of the left testicle, for which he had been treated with surgical resection and adjuvant regional radiotherapy.

A bronchial biopsy, which diagnosed the lung cancer, ruled out a metastasis from the seminoma. A chest computed tomography (CT) scan revealed a mass in the left upper lobe, lymphadenopathy in the left hilum and the mediastinum, and two small nodules in the right lower lobe.

A brain CT scan showed an edematous area with no contrast enhancement in the left temporal lobe, but the patient, who had no neurological symptoms and had a normal neurological clinical examination, refused further investigation using magnetic resonance imaging (MRI). An abdominal CT scan and a bone scan were negative for metastases.

The patient was started on intravenous chemotherapy with a combination of carboplatin, etoposide and epirubicin every 28 days, and after three cycles of therapy he was re-evaluated using CT. The chest CT showed a 50% reduction in the mass in the left upper lobe and in the size of the hilar and mediastinal lymphadenopathy. There was no change in the nodules in the right lower lobe, or in the appearance of the abdominal or brain CT scans.

**Figure 1:**
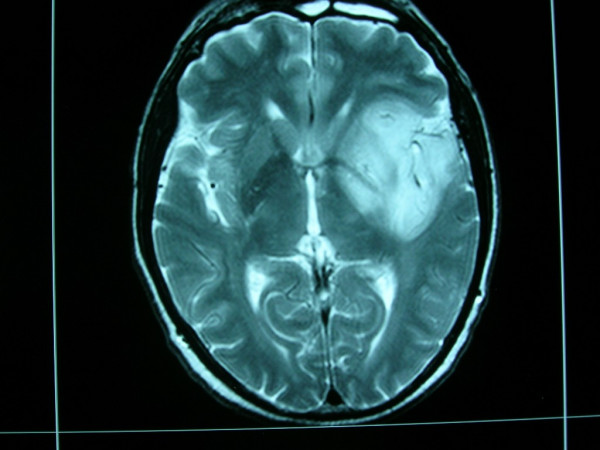
Brain magnetic resonance imaging after the onset of seizures.

Twenty days after the fourth cycle of chemotherapy, the patient was admitted to a neurological clinic because of the onset of self-limiting complex partial seizures, including motionless stare and facial twitching, with no signs of secondary generalization. His relatives stated that, during the previous 2 weeks, the patient had developed neurological symptoms of short-term memory loss and temporary confusion, and behavioral changes including anxiety and depression. He was started on anticonvulsants (Levetiracetam 1500 mg twice daily and alprazolam 1 mg once daily) and soon after underwent a brain MRI, which showed findings of cerebral gliomatosis (Fig. 1).

**Figure 2:**
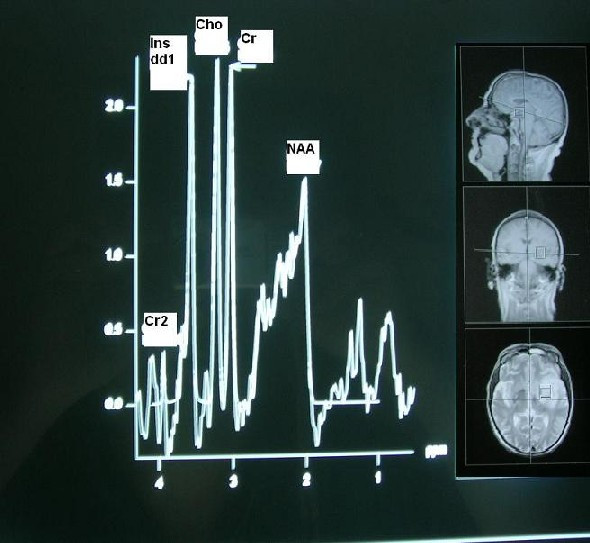
Magnetic resonance spectroscopy of the brain.

**Figure 3:**
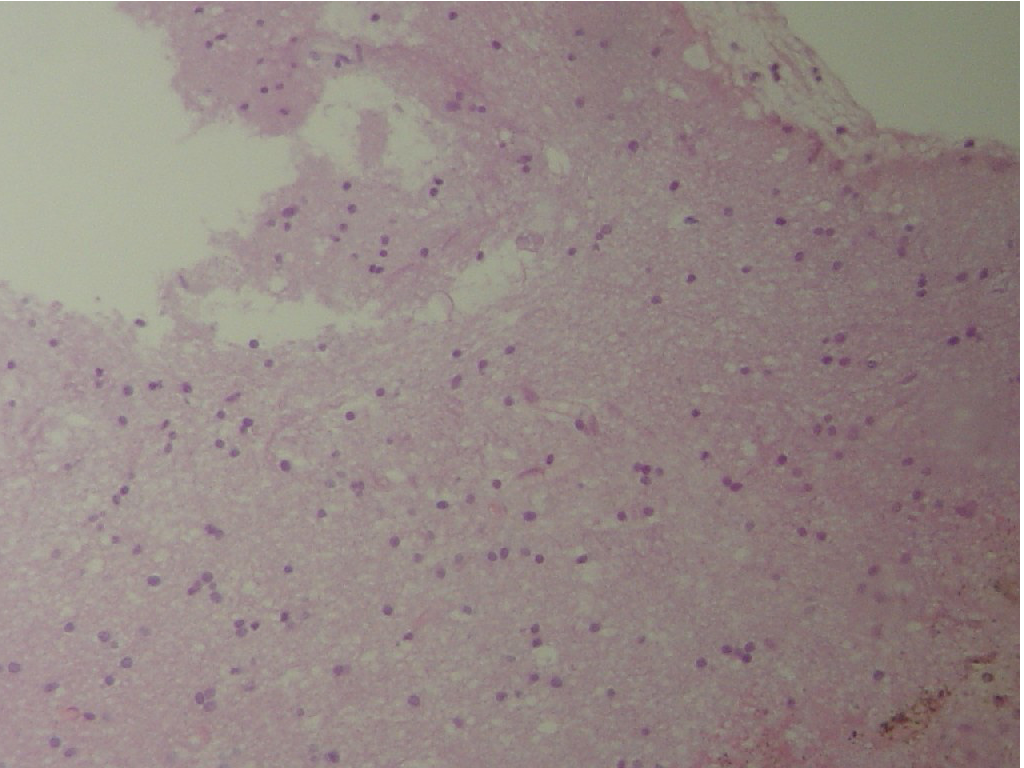
Brain biopsy specimen.

Magnetic resonance spectroscopy of the brain also revealed findings of cerebral gliomatosis (Fig. 2). Clinical and laboratory examinations were not indicative of metabolic, infectious, vascular, drug-induced or chemotherapy-related disease. Serum examination was negative for 'anti-Hu' antibodies. A stereotactic brain biopsy was performed and the pathology specimen revealed brain tissue with areas of lymphocyte infiltration and gliosis, with no evidence of tumor cells (Fig. 3).

Based on the clinical picture, the patient's history of lung cancer, the MRI findings and the results of the brain biopsy, we concluded that our patient had a 'definite' diagnosis of PLE.

The patient continued the anti-epileptic treatment, was started on oral corticosteroids and had two more cycles of chemotherapy, and during this period, he had one more admission for self-limiting simple partial seizures. His neurological status was characterized by occasional self-limiting episodes of short-term memory loss and a temporary confusional state.

After the 6th cycle, the chest CT showed relapse of the disease in the chest.

## Discussion

Paraneoplastic syndromes occur in 10–20% of patients with lung cancer. Small cell lung cancer (SCLC) is associated with the greatest frequency and diversity of paraneoplastic syndromes, including Cushing's syndrome, syndrome of inappropriate antidiuretic hormone secretion and rare paraneoplastic neurological syndromes [1].

The most common paraneoplastic neurological syndromes are Lambert-Eaton myasthenic syndrome and paraneoplastic encephalomyelitis (PEM). Paraneoplastic neurological syndromes are caused by autoimmune processes triggered by cancers and directed against antigens common to both the cancer and the nervous system, designated as onconeural antigens. Autoantibodies against onconeural antigens, strongly associated with cancer and detected by several laboratories in a reasonable number of patients with well defined paraneoplastic neurological syndromes, have been described as 'well characterized' paraneoplastic antibodies [2].

PEM is characterized pathologically by neuronal loss and inflammatory infiltrates in particular areas of the nervous system. The location and severity of the neuronal loss, which may be confined to one area of the nervous system or involve multiple areas over time, predict the clinical symptoms of the patient [3].

The predominant neurological syndrome of PEM at diagnosis is sensory neuropathy. Other common neurological syndromes associated with PEM are cerebellar ataxia, limbic encephalitis (LE), brainstem encephalitis, intestinal pseudo-obstruction and parietal encephalitis.

PLE is clinically characterized by subacute cognitive dysfunction with severe memory impairment, seizures and psychiatric features, including depression, anxiety and hallucinations. Hypothalamic dysfunction may occur, with somnolence, hyperthermia and endocrine abnormalities. PLE may present as an isolated neurological syndrome or as a part of PEM. It may occasionally be associated with thymoma, or testicular, bladder, colon or kidney cancer, or non small cell lung cancer (NSCLC), but SCLC is by far the most frequent underlying tumor. Because LE is associated relatively often with cancer, it is characterized as a 'classical' paraneoplastic neurological syndrome [2].

Clinical diagnosis of PLE associated with lung cancer is difficult. Generally, the following criteria must be fulfilled [3]:

a) clinical picture of seizures, memory loss and/or confusion or psychiatric symptoms suggesting involvement of the temporal lobes or limbic system.

b) temporal relationship (interval of less than 4 years) between the onset of neurological symptoms and diagnosis of lung cancer. PLE usually precedes the diagnosis of cancer by a median time of 8 months, but may also appear after tumor diagnosis, either in the first 6 months (usually associated with progression or relapse of the disease) or when the tumor is in remission (median time 12 months after diagnosis, most commonly associated with tumor relapse).

c) absence of metastatic, metabolic (Wernicke-Korsakoff encephalopathy, sepsis, hepatic or uremic encephalopathy, electrolyte abnormalities), infectious (herpes simplex encephalopathy), vascular (ischemia or hemorrhage), drug-related (drug intoxication or drug withdrawal) or chemotherapy (especially doxifluridine) treatment-related causes that could account for the neurological dysfunction.

d) abnormal MRI of the head characterized by a high-intensity signal on T2-weighted images and atrophy (and sometimes enhancement with contrast injection) on T1-weighted images in one or both medial temporal lobes. In selected difficult cases, co-registration of 18F-fluorodeoxyglucose positron emission tomography may further improve the sensitivity of imaging. CT scans may show no contrast-enhancing lesion in the temporal lobe, but are not sensitive or specific for the diagnosis of PLE.

Other analyses which can help in the diagnosis of PLE are [3]:

a) serum or cerebrospinal fluid (CSF) examination positive for 'anti-Hu' antibodies (autoantibodies generated against the Hu antigen found in the nucleus of neurons). Anti-Hu antibodies have been consistently reported in PLE associated with lung cancer (about 50–60% of patients with SCLC and PLE have anti-Hu antibodies), although their absence does not rule out the syndrome [4]. PLE in anti-Hu-negative patients is more likely to remain isolated throughout the clinical course, whereas patients with anti-Hu antibodies usually develop a multifocal disorder compatible with PEM. Patients with testicular cancer often have anti-Ma2 antibodies (antibodies against Ma2, a protein expressed both in the brain and in testicular tumor tissue). Anti-Ma2 antibodies are also called anti-Ta antibodies. Other autoantibodies occasionally observed in SCLC patients are anti-amphiphysin and anti-CV2 antibodies. All of the aforementioned antibodies are 'well characterized' paraneoplastic antibodies.

b) CSF analysis assists in making the diagnosis of PLE by detecting inflammatory abnormalities (lymphocytic pleocytosis, elevated proteins, intrathecal synthesis of immunoglobulin G, oligoclonal bands) supporting the diagnosis of an inflammatory or immune-mediated neurological disorder and by confirming the absence of malignant cells, excluding (in combination with the absence of meningeal enhancement on the MRI) the presence of leptomeningeal metastases.

c) electroencephalography is useful in assessing whether changes in the level of consciousness or behavior are related to temporal lobe seizures.

d) biopsy from MRI enhancing areas usually reveals perivascular cuffing, interstitial infiltrates of lymphocytes, microglial proliferation, gliosis and neuronal degeneration, and confirms the absence of malignant cells.

In 2004, an international panel of neurologists established diagnostic criteria that divide patients with a suspected paraneoplastic neurological syndrome into 'definite' and 'probable' categories, based on the presence or absence of a typical clinical picture, cancer and specific autoantibodies [2]. According to these criteria, a patient with a typical clinical picture of LE is considered to have a 'definite' PLE when he or she has positive 'well characterized' paraneoplastic antibodies and/or known cancer, if other possible causes of encephalitis have been excluded.

The prognosis of PEM remains poor, in terms of survival and neurological impairment. The median survival time is 11.8 months after onset of the PEM, with a 3-year actuarial survival of 20% [5]. In patients with lung cancer and PEM, the 3-year actuarial survival is 30%. Age and functional status are independent prognostic factors for survival. Effective treatment of the tumor is an independent predictor for symptom stabilization or improvement in PEM.

Patients with PLE and lung cancer who are negative for anti-Hu antibodies seem to improve more often after treatment of their cancer than those who have anti-Hu antibodies. The Anti-Hu(+) patients usually die from complications of their neurological status, whereas in the Anti-Hu(-) group, death is due to progression of the lung cancer. The limited stage of the disease, irrespective of anti-neuronal antibody status, is associated with neurological improvement after tumor treatment.

The treatment of LE is generally unsatisfactory. There are two aspects of the treatment [6]:

a) removing the antigen source by treating the underlying malignancy.

b) suppressing the immune reaction with the use of immunosuppressive drugs or therapies.

The treatment of the underlying malignancy appears to be more effective on neurological outcome than the use of immune modulation. In a study of 200 patients with anti-Hu-associated PEM [5], there was improvement or stabilization of PEM in 37.5% of the patients treated with anti-neoplastic therapy (with or without immunotherapy) in 20.6% of patients treated with immunotherapy and in 11.6% of untreated patients.

Although immunotherapy alone is probably not effective in the majority of the patients, a trial with it should be considered when anti-neoplastic treatment is not possible because the tumor cannot be found or when PEM appears during or after tumor treatment. There are no established protocols for immunotherapy. Immunotherapies include methylprednisolone, azathioprine, tacrolimus, intravenous immunoglobulins +/- cyclophosphamide or methylprednisolone, plasma exchange and removal of plasma IgG by immunoadsorption with a protein A column.

Although there is no generally effective treatment for all such patients, early diagnosis and treatment of the tumor seem to give the best chance to stabilize the disease, especially in patients negative for anti-Hu antibodies [7]. For this reason, when PEM is suspected in a patient without a known malignancy, an intense investigation must be carried out to look for the presence of an associated tumor.

## Conclusion

Paraneoplastic limbic encephalitis is a possible cause of seizures in patients with lung cancer. New onset of paraneoplastic limbic encephalitis in patients with already diagnosed lung cancer is usually associated with progression or relapse of the disease. Patients with paraneoplastic limbic encephalitis and lung cancer who are negative for anti-Hu antibodies are more likely to improve after treatment of the tumor and have lower chances of developing paraneoplastic encephalomyelitis than those who have anti-Hu antibodies. Early diagnosis and treatment of the tumor offer the best chances for improvement in patients with paraneoplastic limbic encephalitis.

## Competing interests

The authors declare that they have no competing interests.

## Authors' contributions

VV and EM drafted the final version of this manuscript. CG helped to draft the manuscript. AK participated in data collection and treated the patient. CG conducted the brain biopsy. SP collected the histological photos and rendered an interpretation. EV evaluated the radiological findings. PC conceived the study and participated in its design and coordination. All authors read and approved the final manuscript.

## Consent

Written informed consent was obtained from the patient for publication of this case report and any accompanying images. A copy of the written consent is available for review by the Editor-in-Chief of this journal.

## References

[B1] Honnorat J Antoine JC Paraneoplastic neurological syndromes Orphanet J Rare Dis 2007 42 22 10.1186/1750-1172-2-22 PMC1868710 17480225

[B2] Graus F Delattre JY Antoine JC Dalmau J Giometto B Grisold W Honnorat J Smitt PS Vedeler Ch Verschuuren JJ Vincent A Voltz R Recommended diagnostic criteria for paraneoplastic neurological syndromes J Neurol Neurosurg Psychiatry 2004 75 1135 1140 10.1136/jnnp.2003.034447 15258215 PMC1739186

[B3] Gultekin S Rosenfeld M Voltz R Eichen J Posner J Dalmau J Paraneoplastic limbic encephalitis: neurological symptoms, immunological findings and tumor association in 50 patients Brain 2000 123 1481 1494 10.1093/brain/123.7.1481 10869059

[B4] Alamowitch S Graus F Uchuya M Rene R Bescansa E Delattre JY Limbic encephalitis and small cell lung cancer. Clinical and immunological features Brain 1997 120 923 928 10.1093/brain/120.6.923 9217677

[B5] Graus F Keime-Guibert F Rene R Benyahia B Ribalta T Ascaso C Escaramis G Delattre JY Anti-Hu-associated paraneoplastic encephalitis: analysis of 200 patients Brain 2001 124 1138 1148 10.1093/brain/124.6.1138 11353730

[B6] Munshi S Thanvi B Chin SK Hubbard I Fletcher A Valliance T Paraneoplastic limbic encephalitis – case report and review of literature Age Ageing 2005 34 190 193 10.1093/ageing/afi017 15713869

[B7] Beukelaar J Smitt P Managing paraneoplastic neurological disorders Oncologist 2006 11 292 305 10.1634/theoncologist.11-3-292 16549814

